# Postoperative mediastinitis after cardiac surgery caused by *Mycoplasma hominis*: a case report

**DOI:** 10.1186/s40792-021-01326-0

**Published:** 2021-11-23

**Authors:** Hiroki Kitagawa, Haruna Shimizu, Keijiro Katayama, Kayoko Tadera, Toshihito Nomura, Kietaro Omori, Norifumi Shigemoto, Taiichi Takasaki, Hiroki Ohge, Shinya Takahashi

**Affiliations:** 1 Department of Surgery, Graduate School of Biomedical and Health Sciences, Hiroshima University, 1-2-3 Kasumi, Minami-ku, Hiroshima, 734-8551 Japan; 2 Department of Infectious Diseases, Hiroshima University Hospital, 1-2-3 Kasumi, Minami-ku, Hiroshima, 734-8551 Japan; 3 Project Research Center for Nosocomial Infectious Diseases, Hiroshima University, 1-2-3 Kasumi, Minami-ku, Hiroshima, 734-8551 Japan; 4 Section of Infection Diseases Laboratory, Department of Clinical Support, Hiroshima University Hospital, 1-2-3 Kasumi, Minami-ku, Hiroshima, 734-8551 Japan; 5 Division of Clinical Laboratory Medicine, Hiroshima University Hospital, 1-2-3 Kasumi, Minami-ku, Hiroshima, 734-8551 Japan; 6 Translational Research Center, Hiroshima University, 1-2-3 Kasumi, Minami-ku, Hiroshima, 734-8551 Japan

**Keywords:** *Mycoplasma hominis*, Postoperative infections, Cardiovascular surgery, Molecular identification, MALDI-TOF MS

## Abstract

**Background:**

*Mycoplasma hominis* is a human commensal bacterium of the urogenital tract, and extragenital infection caused by *M. hominis* has rarely been reported. The identification of *M. hominis* is challenging, and surgeons are generally not aware that this bacteria can cause postoperative infection. Here, we report a rare case of postoperative mediastinitis caused by *M. hominis* after cardiac surgery in an immunocompetent patient.

**Case presentation:**

A 54-year-old man presented with pain and purulent discharge from the wound after aortic valve replacement and patent foramen ovale closure. However, Gram staining and culture of bacteria from the purulent discharge was negative, and empiric sulbactam/ampicillin therapy was not effective. This patient developed mediastinitis and rupture of a pseudoaneurysm of the ascending aorta caused by mediastinitis, and re-operation was performed. Then, postoperative mediastinitis caused by *M. hominis* or *Ureaplasma* species was suspected and bacterial cultures targeting these pathogens were performed. *M. hominis* was identified from abscess and tissue obtained from the surgical site and urine. A final diagnosis of postoperative mediastinitis caused by *M. hominis* was determined. The patient was initially treated with levofloxacin and then with minocycline for 3 weeks. The patient’s clinical condition improved; the patient was transferred to another hospital.

**Conclusion:**

The role of *M. hominis* as a cause of postoperative infection might be underestimated in cardiac surgery. *M. hominis* should be considered when culture-negative purulent discharge is observed or there is no response to standard empiric treatment of postoperative infections.

## Introduction

*Mycoplasma hominis* is a human commensal bacterium of the urogenital tract that is prevalent in sexually active adults [1] and typically causes urogenital infections [1, 2]. *M. hominis* rarely causes non-urogenital postoperative infection, including post-transplant infection after kidney, lung, and heart transplantation [3–5], mediastinitis after cardiac surgery [6], septic arthritis after joint replacement [7], and meningitis or brain abscess after neurosurgery [8, 9]. *M. hominis* cannot be detected by Gram staining, and its identification using conventional microbiological identification techniques is challenging. In addition, *M. hominis* is intrinsically resistant to antimicrobial agents such as beta-lactams, which are usually used during the perioperative period, because this organism lacks a cell wall. Because of these characteristics, postoperative infections caused by *M. hominis* might be underestimated, and the prevalence of postoperative mediastinitis caused by *M. hominis* is currently unknown. Here, we report a rare case of postoperative mediastinitis caused by *M. hominis* after cardiac surgery in an immunocompetent patient.

## Case report

**Fig. 1:**
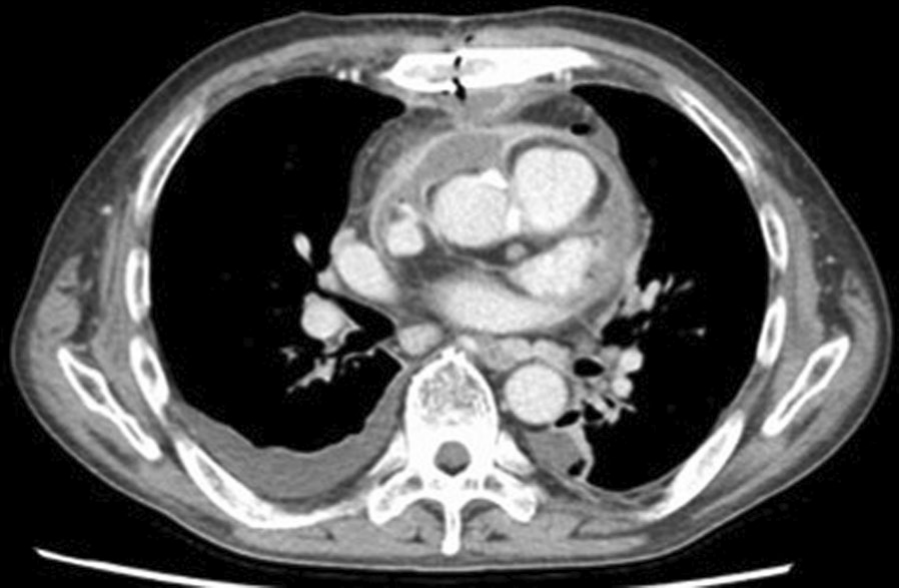
Computed tomography showing retrosternal fluid and free air, indicating mediastinitis

**Fig. 2:**
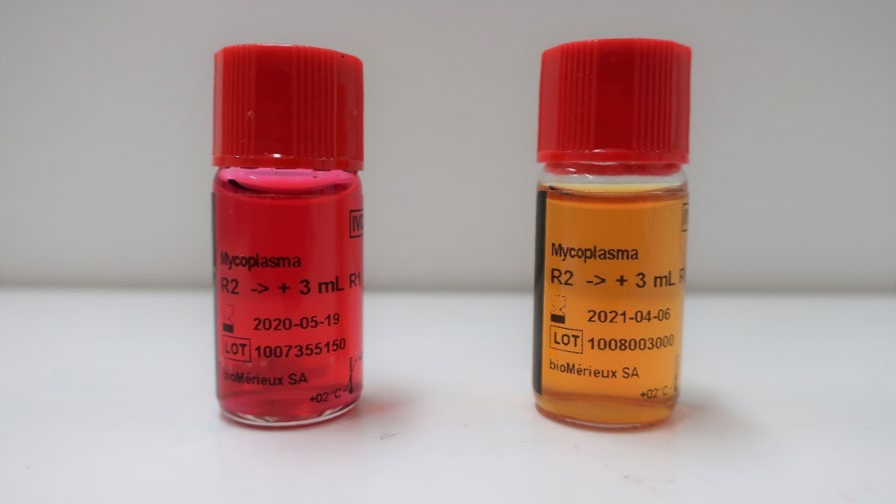
The broth in which color changed from yellow to red (left) and the control broth (right)

A 54-year-old man with severe aortic valve stenosis and patent foramen ovale (PFO) was hospitalized in our institution to undergo aortic valve replacement with a biological valve and PFO closure. This patient had a medical history of Ménierè syndrome and underwent surgery with prophylactic administration of cefazolin. The patient exhibited pain in the region of the surgical site, and purulent discharges from the wound were observed on postoperative day (POD) 11. Plain computed tomography (CT) showed no signs of postoperative mediastinitis such as retrosternal fluid and free air. Therefore, at that point, mediastinitis was not suspected. Blood culture and purulent discharges were submitted to culture, and sulbactam/ampicillin (3 g every 8 h) administration was empirically started. Microscopic examination of the purulent discharges showed no bacteria on Gram staining; however, many neutrophil aggregations were observed. The bacterial culture of purulent discharges and blood culture using the BacT/ALERT 3D system (bioMérieux, Marcy l’Etoile, France) was negative. The patient developed fever, and purulent discharges from the wound were observed despite the administration of sulbactam/ampicillin. On POD15, enhanced CT showed the appearance of a retrosternal fluid and free air, and mediastinitis was suspected (Fig. 1). Then, re-opening and vacuum-assisted closure for the sternotomy site was started. On POD16, the patient suddenly developed cardiopulmonary arrest, and transthoracic echocardiography showed cardiac tamponade. Extracorporeal membrane oxygenation (ECMO) support was started, and the patient was moved to the operating room to reopen the chest. Bleeding from the ascending aorta was detected, and rupture of a pseudo-aneurysm caused by mediastinitis was diagnosed. The pseudo-aneurysm originated primarily from the suture lines of a former aortic valve surgery. In addition, the infected aortic wall adjacent to the suture line had become vulnerable. The ascending aorta was repaired using a bovine pericardial patch, and debridement and omentopexy for mediastinitis was performed. The antimicrobial agent was changed to meropenem (1 g every 12 h) and vancomycin after surgery. The dose of antimicrobial agents was reduced due to renal dysfunction. Gram staining of the abscess and tissue sampled from the surgical site did not show any visible microorganisms; however, many neutrophil aggregations were observed. The cardiovascular surgeon consulted the department of infectious disease, and the infectious disease expert suspected postoperative mediastinitis caused by *M. hominis* or *Ureaplasma* species. On POD18, to identify *M. hominis* and *Ureaplasma* species, abscess and tissue obtained from POD16 and urine obtained on POD17 were cultured using urea-arginine LYO2 broth (bioMérieux). Then, the antimicrobial therapy was changed from meropenem and vancomycin to levofloxacin (250 mg every 24 h) and vancomycin. A color change in the broth was observed 24–48 h after the start of culture (Fig. 2). DNA was extracted from the broth using a MORA-EXTRACT DNA extraction kit (Kyokuto Pharmaceuticals Industrial Co., Ltd., Tokyo, Japan), and polymerase chain reaction (PCR) using *M. hominis*, *Ureaplasma paruvum*, and *Ureaplasma urealyticum*-specific primers was performed [10, 11]. All samples were positive for *M. hominis*, and a final diagnosis of postoperative mediastinitis caused by *M. hominis* was determined. No growth of other bacteria was detected from the abscess, and therefore, the antimicrobial agent was changed to only levofloxacin on POD21. The two sets of blood cultures sampled on POD16 were negative. The abscess, tissue, and urine samples were also cultured on Brucella HK agar plates (Kyokuto Pharmaceutical Industrial Co., Ltd.) under anaerobic conditions at 35 °C. After 4 days of anaerobic culture, numerous pinpoint colony formations became visible on the Brucella HK agar plates. *M. hominis* was identified from the colonies by PCR using *M. hominis*-specific primers. Matrix-assisted laser desorption/ionization time-of-flight mass spectrometry (MALDI-TOF MS) analysis was also performed with a BD MALDI Biotyper sirius system (Becton, Dickinson and Company, USA) using the MBT Compass 4.1 with reference database MBT Compass library: Ver.9.0.0.0 (8468MSPs) (Bruker Daltonik GmbH, Bremen, Germany). MALDI-TOF MS analysis using pure culture colonies, performed by direct transfer methods as previously described [12], identified the isolates as *M. hominis* with a high score value ≥ 2.000.

The minimal inhibitory concentration (MIC; μg/mL) values of clindamycin, levofloxacin, ciprofloxacin, and minocycline were determined using the E-test (bioMérieux) (Table 1). However, these MIC values were for reference only because the interpretation of MICs determined by the E-test is not mentioned in the criteria of Clinical and Laboratory Standard Institute (CLSI) M43-A [13].

**Table 1:** Minimum inhibitory concentration of the *Mycoplasma hominis* isolate

Antimicrobial agents	MIC (µg/mL)
Clindamycin	0.023
Levofloxacin	0.023
Ciprofloxacin	0.047
Minocycline	0.016

MIC, minimal inhibitory concentration

After surgery, the postoperative course of mediastinitis was good. However, stenosis of the ascending aorta at the repair site was observed using transesophageal echocardiograph which showed that the maximum flow velocity at the stenosis site was 5.1 m/s. This patient could not be weaned from ECMO due to the stenosis. On POD23, thoracic endovascular aortic repair was performed for stenosis of the ascending aorta, and the intra-vertical hematoma was removed by re-opening the chest. After surgery, the patient was able to be weaned off ECMO. Although an abscess was not detected at the surgical site, the hematoma was cultured in urea-arginine LYO2 broth, and the broth changed color after 24 h. In addition, PCR using *M. hominis*-specific primers was positive. Although the MIC of levofloxacin determined by the E-test was not high, we suspected that the *M. hominis* isolate was resistant to levofloxacin, and the antimicrobial agent was changed to minocycline (100 mg every 12 h) on POD25. After re-surgery and changes to the antimicrobial agent, the postoperative course was good. Antimicrobial treatment with minocycline was continued for 3 weeks and the patient’s clinical condition improved; the patient was transferred to another hospital on POD80.

## Discussion

We report a rare case of postoperative mediastinitis caused by *M. hominis* after cardiac surgery in an immunocompetent patient. Postoperative mediastinitis caused by *M. hominis* has rarely been reported, and Le Guern et al. reviewed 17 cases [6]. The median age was 55 years, and all reported patients were male. The median onset of clinical symptoms was 14 days after surgery. These clinical features were consistent with this patient. *M. hominis* colonizes the human urogenital tract in sexually active adults [1] and the respiratory tract, but this is less frequent [14]. Although a clear origin of the infection is difficult to recognize, it was hypothesized that invasive medical procedures such as urinary catheterization lead to bloodstream invasion of *M. hominis* and seeding of the surgical site [15]. In addition, recent reports have shown donor-derived *M. hominis* infection in lung transplant recipients [4]. In this study, the patient was not immunocompromised and urine culture was positive for *M. hominis*, but the septum was negative. Therefore, *M. hominis* colonized the urinary tract, and urinary catheterization during surgery might lead to bacteremia and seeding of the surgical site. As *M. hominis* is frequently found in the human urogenital tract [1] and catheterization is a common procedure during surgery, the possibility of postoperative *M. hominis* infections could be underestimated.

The identification of *M. hominis* infection is often challenging due to the slow growth of the colonies and the absence of a cell wall, which gives a negative Gram stain result. Therefore, it is difficult to detect *M. hominis* using standard microbiological methods without first suspecting them as a cause of postoperative infections. In addition, cardiovascular surgeons are generally not aware of the fact that *M. hominis* can cause postoperative infection. In this study, the infectious disease expert suspected *M. hominis* or *Ureaplasma* spp. because Gram staining and culture of the purulent discharges obtained from the surgical site were negative and sulbactam/ampicillin was not effective. In addition, Gram staining of the abscess and tissue sampled from the surgical site of re-operation was negative. To identify *M. hominis,* we used urea-arginine LYO2 broth and anaerobically cultured samples on Brucella HK agar plates for 4 days. As it is not popular to use urea-arginine LYO2 broth in clinical microbiological laboratories of general hospitals in Japan, it may be better to extend the culture period under 5% CO_2_ on blood agar plates or under anaerobic condition on Brucella HK agar plates to identify *M. hominis* because of its slow-growing nature. The identification of *M. hominis* is usually performed by 16S ribosomal DNA sequencing, PCR using *M. hominis*-specific primers, and MALDI-TOF MS [6, 9, 10]. In this study, we used PCR with *M. hominis*-specific primers and MALDI-TOF MS. Although MALDI-TOF MS has been reported to be a useful tool for the identification of human *Mycoplasma* species including *M. hominis* [16], *M. hominis* has not been included in the clinical use MALDI Biotyper database. In this case, we found that MALDI-TOF MS analysis performed by a MALDI Biotyper system with the reference database MBT Compass library: Ver.9.0.0.0 (8468MSPs) was very useful for the rapid identification of *M. hominis*. Because of its slow growth, 16S ribosomal DNA sequencing or PCR with *M. hominis*-specific primers from positive urea-arginine LYO2 broth might be faster than MALDI-TOF MS using bacterial colonies. Previous reports showed that 16S rDNA was sequenced directly from clinical samples and is a good method to identify *M. hominis* [6]. Further study is needed to evaluate if MALDI-TOF MS can directly identified *M. hominis* from positive urea-arginine LYO2 broth and positive blood culture bottles, as well as a pure cultured colony.

Postoperative mediastinitis is a major complication of cardiac surgery, with a low incidence but serious consequences in terms of morbidity and mortality [17]. Early diagnosis can lead to the early use of appropriate antimicrobial agents for *M. hominis* and avoid repeated surgical interventions. However, the identification of *M. hominis* is challenging under conventional microbiological identification techniques without suspecting them. Therefore, if a patient develops unexplained postoperative fever in cases of otherwise culture-negative infections, particularly if treated with beta-lactam antibiotics, and has a poor response, it is important to consider *M. hominis* infection as a differential diagnosis. When clinicians suspect *M. hominis*, they should inform the clinical microbiological laboratory and request to extend the culture period.

Beta-lactam antibiotics are generally used for antimicrobial prophylaxis in cardiac surgery [18]. In addition, broad-spectrum beta-lactam antibiotics and glycopeptides are usually used for empiric treatment of postoperative infection after cardiac surgery targeting gram-positive cocci, including methicillin-resistant *Staphylococcus aureus* and gram-negative bacilli [17]. However, beta-lactam antibiotics and glycopeptides are not effective against *M. hominis* because of the absence of a cell wall. *M. hominis* is generally susceptible to tetracyclines, clindamycin, and fluoroquinolones, but intrinsically resistant to clarithromycin and erythromycin [13]. *M. hominis* isolates resistant to these antimicrobial agents have also been reported; however, the resistance rate to fluoroquinolone, tetracyclines, and clindamycin of *M. hominis* varies by report [19–21]. In this case, we started levofloxacin after surgical debridement; however, the culture of the intra-vertical hematoma after 7 days of levofloxacin treatment was positive for *M. hominis*. Although the MIC of levofloxacin determined using the E-test was not high, we suspected that *M. hominis* was resistant to levofloxacin, and the antimicrobial agent was changed to minocycline. This condition might be associated with the short treatment duration of levofloxacin or residual intra-vertical hematoma. CLSI M43-A indicates that agar disk diffusion is not useful for testing mycoplasmas because there has been no correlation between inhibitory zones and MICs [13]. In addition, the method using the E-test was not mentioned in CLSI M43-A. However, broth microdilution and agar dilution, which are recommended methods to determine MICs by the CLSI M43-A, are not practical in clinical microbiological laboratories in Japanese hospitals. Therefore, the careful observation of clinical course after the administration of antimicrobial agents for *M. hominis* is needed.

## Conclusion

We report a rare case of postoperative mediastinitis after cardiac surgery caused by *M. hominis* in an immunocompetent patient. The role of *M. hominis* as a cause of postoperative infections might be underestimated in cardiac surgery. *M. hominis* should be suspected*,* especially when culture-negative postoperative infections are observed or there is no response to standard empiric treatment of postoperative infections.

## Data Availability

Not applicable.

## Abbreviations

CLSI: Clinical and Laboratory Standard Institute
CT: Computed tomography
MALDI-TOF: Matrix-assisted laser desorption/ionization time-of-flight mass spectrometry
MIC: Minimum inhibitory concentration
PFO: Patent foramen ovale
POD: Postoperative day
